# CBRAM technology: transition from a memory cell to a programmable and non-volatile impedance for new radiofrequency applications

**DOI:** 10.1038/s41598-022-08127-x

**Published:** 2022-03-08

**Authors:** Sergio López-Soriano, Jayakrishnan Methapettyparambu Purushothama, Arnaud Vena, Etienne Perret

**Affiliations:** 1 Laboratoire de Conception et d’Intégration des Systèmes, Grenoble INP, Université Grenoble Alpes, 26902 Valence, France; 2 Institut d’Electronique et Systèmes (IES), Université de Montpellier/CNRS, 34095 Montpellier, France; 3 Present Address: Department of Communications and Information Technology, Universitat Pompeu Fabra, 08018 Barcelona, Spain; 4 Present Address: School of Engineering and Physical Sciences, Heriot-Watt University, Edinburgh, EH14 4AS UK

**Keywords:** Electrical and electronic engineering, Characterization and analytical techniques

## Abstract

Electrical resistance control programming of conductive bridging random access memory (CBRAM) radio frequency (RF) switches could benefit the development of electronically controlled non-volatile RF attenuators and other reconfigurable devices. The object of this study is to adapt a conventional CBRAM based memory cell to be used as an RF switch, and to demonstrate the feasibility of programming non-volatile RF CBRAM switches to achieve specific target resistances within a range of continuous values. The memory-RF technologic transition implies a drastic increase of the geometry in order to handle a much higher power, a decrease of the transition capacitance in order to operate at much higher frequencies, and a decrease of the LRS to a few ohms, which is critical for RF applications. These studies are initially performed on an in-house made RF CBRAM cell array at DC frequency, and then extended successfully to a co-planar waveguide (CPW) based shunt mode RF switch with an integrated CBRAM cell. Reliability of the proposed technique is validated through detailed analysis of factors like repeatability of the process, time stability of programmed states, and statistics of time taken to converge to a desired resistance value for an arbitrary RF CBRAM switch.

## Introduction

Conductive bridging random access memory (CBRAM) is a technology initially introduced as a memory technology^1–4^ and was later identified to be an efficient RF switching solution in the recent decade^5–11^. Peculiar properties of CBRAM switches in comparison to classic RF switching solutions available at present, like solid-state semiconductor based, and MEMS solutions, include non-volatility, ease of fabrication due to suitability of process steps to an industrial roll-to-roll process, printability^12^, and similar^13^.

Typically for memory applications, there is not any stringent requirement of impedance magnitudes, other than a notable distinction between low resistance state (LRS) and high resistance state (HRS), in the order of a few thousands of ohms, which is well acceptable^4,14^. On the other hand, for RF switching applications, it is necessary that the LRS should always be minimum, in the order of a few ohms (say < 5 Ω), and the HRS should be of the order of a few hundreds of ohms (say > 1 kΩ), to respectively facilitate excellent RF transmission and isolation behaviour with minimum ohmic losses^7,9,13^.

One of the interesting properties of CBRAM switches is the potential possibility of modifying the cell resistance (*R*) value through control over the filament dimensions^15^. Thus, instead of having a CBRAM with two states (classic high or low states for memory, or ON or OFF states in RF applications), it is expected to be able to parameterize the CBRAM so that it can have a number of discrete resistances among the LRS and HRS values^16–18^. These approaches rely on fixing the programming pulse parameters, expecting that the cell will be programmed to a resistance value within the desired resistance range. These studies^16–18^ show resistance levels separated by an order of magnitude from each other, which makes multi-level programming a suitable technique for memory applications. However, RF applications require a continuous range of resistance values in order to provide high accuracy when tuning the S-parameters, as it will be demonstrated later in this document.

It is well known that the cell resistance in LRS is mainly a function of the filament conductivity and geometry^4^. In this approach, the conductive properties of the filament are a function of the chosen electrode pairs and the redox mechanism, and it is limited by the number of available natural or engineered material choices^4,12^. On the other hand, it is also well known that the filament geometry, such as its diameter, is dependent on the SET/RESET actuation pulses^6^, associated to filament formation and dissolution processes respectively. Besides, CBRAM filament sizes, and thus resistances, are a function of the magnitude of the applied DC pulses^9,19,20^. Experimental and simulation results of demonstration of such trial and error modification of CBRAM RF switch LRS resistance are reported in^6,12,13^. Therefore, it seems logical to use synthesized DC pulses to achieve the desired resistance values, given specific cell dimensions, electrolyte characteristics, and electrode properties. Then, to really control the resistance value, i.e. to obtain an accurate value over a large impedance range, we have introduced an approach that uses different excitation signals with a feedback loop algorithm, that is to say an iterative approach so that the resistance value of the CBRAM gradually converges towards the expected value. Indeed, it has been observed that the filament formation process is non-deterministic, i.e., the filament shape changes between different realizations, and consequently the resistance of the cell can also undergo variations that can be fatal for some applications. Therefore, the introduction of an iterative approach that drives the generation of different excitation signals is an attractive solution to the problem, one that has never been implemented before.

**Figure 1:**
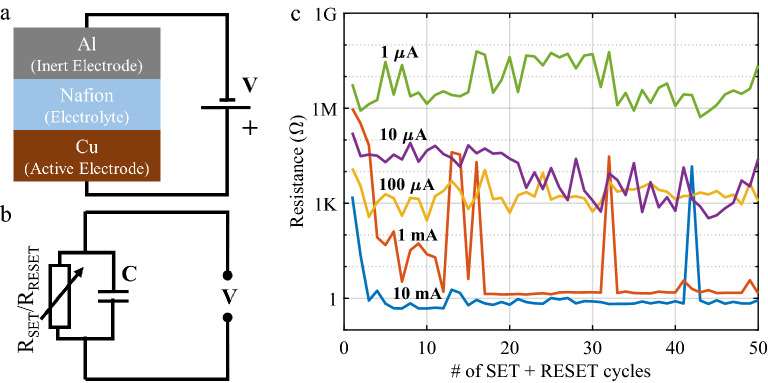
(**a**) Schematic of the MIM stack. (**b**) Equivalent circuit. (**c**) Test results of 50 consecutive SET-RESET operations each on five different cells with current limits Ilim = 10 mA, Ilim = 1 mA, Ilim = 100 µA, Ilim = 10 µA and Ilim = 1 µA, SET triangular pulse sweep rate = 2.8 V/s and RESET rectangular pulse width = 1 s.

Till date, the limits of multilevel programming, in terms of the maximum number of levels that a CBRAM cell can be programmed to, haven’t been quantified. The following experiment is conducted in order to give the reader a better understanding of this issue. The cell stack used in this experiment, together with its equivalent circuit, are presented in Fig. 1a,b respectively. The switching I–V curve can be found in the Supplementary Note 1 as Supplementary Fig. S1. The fabrication process is detailed later in the methods section. Fifty consecutive switching cycles were applied over five different fresh CBRAM cells. Each cycle consists of one SET and one RESET pulse. The SET pulses consist of 16 V triangular pulses with five different current limits (this is a typical fail-proof choice from our previous realizations^6^). And the RESET pulses consist of rectangular pulses of − 20 V and current limit of 100 mA. It should be noted that these voltages are higher than those typically used for memory applications, but are still fully compatible with RF applications where, depending on the technology, voltages up to 40 V can be used to command RF switches^21,22^. The results of the experiment are shown in Fig. 1c. The values correspond to measurements taken after the application of each SET pulse. This experiment demonstrates that the cell resistance varies considerably from one cycle to the other, highlighting that the random nature of the RF CBRAM cell resistance, which is difficult to control with the classic approach of using switching pulses of constant voltage and current values. The relative standard deviations (RSDs) for the five realizations under test were RSD10 mA = 130.9%, RSD1 mA = 136.1%, RSD100 µA = 98.9%, RSD10 µA = 482.9% and RSD1 µA = 635.7%. This huge variability drastically reduces the number of possible programming levels but the results show an even more harmful effect. In fact, the resistance values corresponding to the 1 mA and 10 mA cycles show that, occasionally, the RF CBRAM cell cannot be set by applying a SET pulse, thus retaining a HRS, which is several orders of magnitude higher. As a consequence, the cell resistance value falls into ranges considered as a different programming level. These events actually cross several programming levels, therefore producing errors impossible to track, identify or correct. This behaviour is different from that observed in CBRAM cells for memory applications, where a more direct link exists between the amplitude of the current used to program the cell and the value of the resistance obtained^23^.

Hereafter, we present a novel and innovative approach towards resistance programming techniques for CBRAM RF switches, based on an in-house developed heuristic algorithm, to modulate the actuation control pulses applied to CBRAM RF switches. In this contribution, we propose a resistance programming technique that is able to program RF CBRAM cells to any desired resistance, over a continuous range of values, in opposition to multilevel programming, even for very low values in the order of a few ohms.

### Main differences between CBRAM cells for RF and memory applications

This section is intended to clarify some unavoidable differentiations between designing CBRAM switches for RF applications and classical memory applications (which is the parent filed of this technology). The main differences^4,24,25^ are summarized in Table 1. From the RF application perspective, the accuracy and range of LRS, and OFF (RF) state capacitance are the most important factors. Indeed, the figure of merit (FOM) of RF switches is generally expressed in frequency such as^5^ f_CO_ = (2π·R_ON_·C_OFF_)^−1^, and the main range of interest for R_ON_ is between 0 and 100 Ω for reconfigurable RF components, and below 3 Ω for conventional RF switches. Whereas in memory application only a notable distinction among the LRS and HRS would do the job to indicate two distinct memory states, with a few kΩ at LRS.

**Table 1:** Brief comparison of performance/design parameters of CBRAM technology as an RF switch and as a memory.

CBRAM technology application	RF switch	Memory
Resistance requirements	Low resistance states (LRS) should be necessarily less than a few ohms (e.g. < 100 Ω) and high resistance states (HRS) states should be greater than few kΩ to ensure adequate RF transmission and isolation^24^	LRS and HRS states require only a notable distinction. A few kΩ at LRS (This is at least 100 times greater than what is desired for an RF switch application) and a few MΩ at HRS states are well acceptable^4^
Cell size	Cell size (feature size) of the order of micrometres. This is a trade-off, principally among two factors (a) The off-state capacitance of the switch: larger the cross-section area of electrodes facing each other, larger the off-state capacitance—we try to keep this low (b) For an RF switch unlike the memory, a few hundreds of mA of current handling are desired, even though a pin-point study is not done in this work, a larger feature size is desired to enhance this performance and prevent any thermal breakdown. In the presented case, the approximate cross section area of the electrodes (facing each other in an overlap) is 100 μm × 300 μm, and the ion-conductor thickness is 600 nm In addition, in our studies, we try to manufacture the switch using ‘clean-room less’ technologies using instrumentation compatible with industrial mass production and this naturally contributes to a slightly larger feature size than in ‘clean-room’ based techniques^6,24^	Cell size (feature size) of the order of nanometres. As an example the memory devices presented in Refs.^4,23^ have an electrode surface area of the order of < 5 μm^2^ and ion-conductor thickness of < 50 nm
Repeatability	Repeatability of LRS states with a good precision and accuracy to low resistance values are critical. Here, tolerance of LRS is more critical, than for the HRS	Repeatability of LRS and HRS states with a good separation among each are critical. In memory application the specific values of LRS and HRS are not critical as long as they fall in a given tolerance range (which is well higher than for RF switch applications)
Reliability	Reliability and repeatability as a single stand-alone switching device is very critical. It is not easy to bypass an RF path if a dedicated CBRAM switch goes faulty in a given RF switch topology	Reliability as a group/memory block is important. A faulty CBRAM cell could be bypassed in a group without much impact on the overall efficiency of the memory block
Critical requirements	Linearity, power handling capability, and switching speed are critical	Read–write speed is critical

The differences between RF and memory CBRAM cells described in Table 1 push towards the requirement of a dedicated manufacturing process and unique programming techniques to achieve a precision resistance control for CBRAM cells for RF applications, as addressed herewith in this study.

### Precision resistance programming algorithm (PRPA)

In view of the previously presented facts, an automatized process that can synthesize adaptive programming pulses to achieve any target resistance will provide a more accurate control of the cell resistances. For instance, such a dynamic approach would be able to select a different strategy when the cell resistance crosses the target resistance value and it should also be able to recognize when the target resistance has been reached. In addition, such algorithm should also provide a way to reverse the filament formation process autonomously.

**Figure 2:**
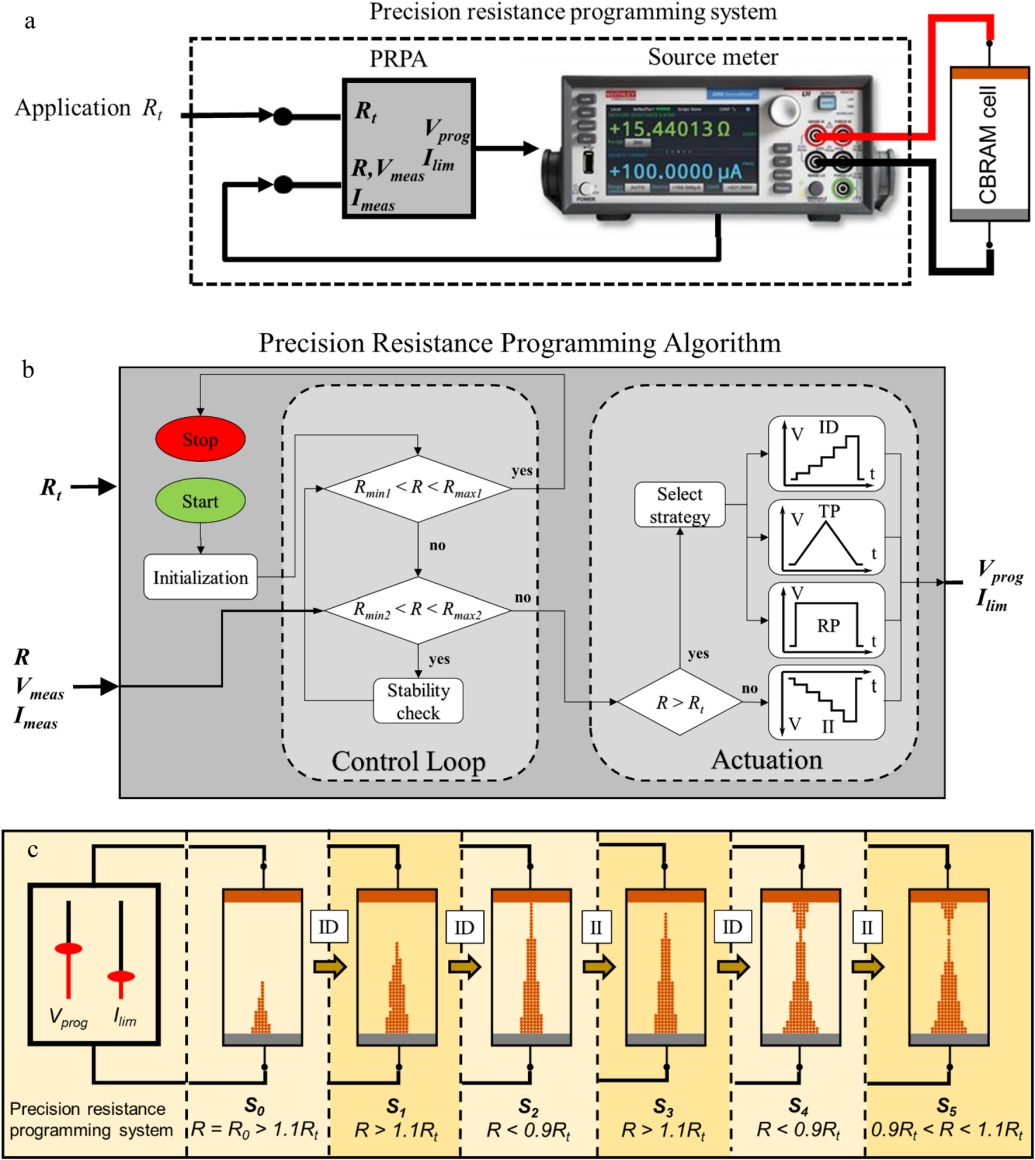
(**a**) Schematic of the measurement/actuation system. (**b**) Precision resistance programming algorithm flowchart. (**c**) Example of a cell programming using the proposed precision resistance programming algorithm (PRPA).

The PRPA consists of an iterative procedure that implements a comparator, that checks the cell resistance at every step, and a logical actuator, that selects the parameters of the next voltage pulse. The PRPA communicates with a source meter which generates specific pulses and applies them to the cell terminals. A schematic of the system is depicted in Fig. 2a. *R* and *R*_*t*_ stand for the measured cell resistance and the target resistance respectively. The tuning parameters used to program the CBRAM cells are the selected voltage level *V*_*prog*_ (V) and the maximum output current *I*_*lim*_ (A). Once the programming pulses are triggered, the output voltage and current are measured as *V*_*meas*_ and *I*_*meas*_ respectively. The following equations fully explain the macroscopic behaviour of the CBRAM cell for a voltage source programming:

1 $${\text{If}}\left( { \, \frac{{V_{prog} }}{R} \le I_{lim} } \right) \, \Rightarrow \, V_{meas} = V_{prog} \Rightarrow I_{meas} = \frac{{V_{prog} }}{R} < I_{lim} ,$$

2 $${\text{If}}\left( {\frac{{V_{prog} }}{R} > I_{lim } } \right) \Rightarrow I_{meas} = I_{lim } \Rightarrow V_{meas} = I_{lim } \cdot R < V_{prog} .$$

The PRPA flowchart is depicted in Fig. 2b. It consists of two main blocks, namely the control loop and the actuation logic. The control loop function is to ensure that the algorithm reaches an *R* value within the tolerance limits and that this value is stable over time. The stability check (*sc*) consists of a measurement loop using the last *V*_*meas*_ and *I*_*meas*_ values as the new *V*_*prog*_ and *I*_*lim*_ respectively. Once the algorithm reaches the required accuracy and stability, it stops.Four strategies are proposed for the rectification of *R*. Three are used when *R* > *R*_*t*_, and therefore they imply using positive voltage pulses. incrementaldecrease (*ID*) is the default initial strategy for decreasing *R*. Its purpose is to increase the energy absorbed by the active metal gradually by limiting the voltage/current at the cell terminals. triangluarpulse (*TP*) and rectangularpulse (*RP*) strategies are intended to produce large resistance variations, mainly to break out of a steady state or a loop. *TP* is intended to trigger a set operation. This is helpful when *ID* can’t decrease *R* from a HRS. *RP* is used to produce either a set or a unipolar reset^26^ (see Supplementary Note 2), after trying the other strategies without successful programming. When *R* < *R*_*t*_, incrementalincrease (*II*) is used to produce a bipolar reset in a stepwise manner. No recovery behaviours are required to increase the cell resistance because the filament dissolution is easily triggered. After each algorithm iteration, the algorithm checks if the measured resistance value falls within the tolerance boundaries (*R*_*minX*_ < *R* < *R*_*maxX*_, where *X* = {1, 2} for the two different boundaries). If positive, then the PRPA stops, assuming that the cell is successfully programmed. A detailed explanation of the algorithm and the corresponding strategies (including *V*_*prog*_ and *I*_*lim*_ updating instructions) can be found in the “Methods” section. The pseudo-codes for the main routine and the programming strategies are shown in Alg. 1 and Alg. 2.
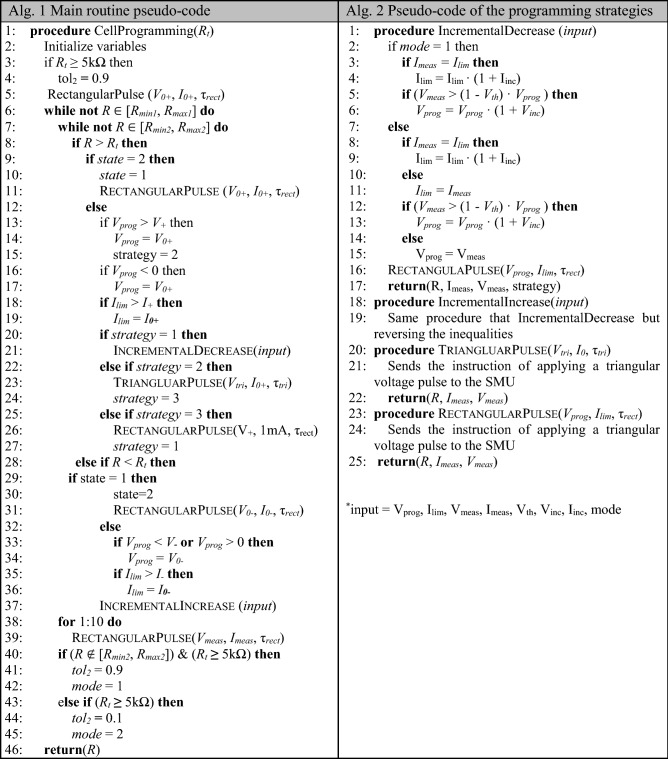


Summing up, the PRPA is an iterative routine in which the operations of increasing and reducing the size of the filament are discretized by the controlling *V*_*prog*_ and *I*_*lim*_, to produce small shifts in the energy delivered to the active metallic particles (Cu), ionizing the copper atoms and enabling the copper ions to travel through the electrolyte (Nafion).

An example of a cell programming operation using such automatized procedure is depicted in Fig. 2c. The example consists of five different steps or transitions denoted by arrows. Each state (*S*_*i*_) is illustrated using a different box. The example starts (*S*_0_) with a random initial resistance *R*_0_. After a measurement showing that *R* > 1.1*R*_*t*_, the procedure selects the *ID* strategy and adjusts the output voltage and current limit to program the cell (*S*_1_). Then the cell resistance is measured again finding that the cell resistance is still over the target, therefore the output parameters are updated. In *S*_2_, the cell resistance turns to be lower than the target range, therefore the algorithm will select the *II* strategy in order to start applying negative voltages to the cell. Consequently, the filament is broken in *S*_3_. In turn, this increases the cell resistance over the target range which results in the application of a positive voltage pulse (*ID*) resulting in a wider filament and a lower cell resistance (*S*_4_). Again, the cell resistance value is below the target range, and so the resistance control tries to increase the cell resistance by applying a negative pulse (*II*). In the last step (*S*_5_), the filament presents a resistance value within the target range. Then the algorithm *sc* procedure is triggered after which, if the cell resistance measured value falls within the tolerance limits, the algorithm stops, else the algorithm continues.

This example is given here to provide a simple explanation of the algorithm and also to demonstrate the need of an iterative process to reach a low programming error (ε). The algorithm may require a different number of steps (*S*_*n*_) for every programming operation. The advantage of this approach is that the resistance value is obtained within the specified range, however the iterative approach does not allow to know a priori the number of iterations it takes to obtain this value and therefore neither the corresponding time.

## Results

### PRPA operation on single cells

**Figure 3:**
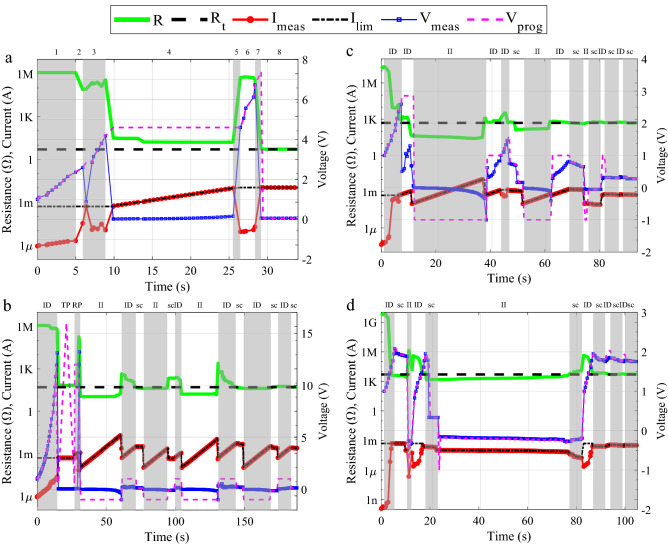
Resistance programming algorithm runs on four different cells. (**a**) Rt = 5 Ω. (**b**) Rt = 50 Ω. (**c**) Rt = 500 Ω. (**d**) Rt = 5000 Ω. In these experiments TP sweep rate was set to 2.8 V/s, RP pulse width was 5 s, II and ID pulse width were set to 0.68 s.

Figure 3a–d show the PRPA execution over four different virgin CBRAM cells. *R*_*t*_ is set to 5 Ω, 50 Ω, 500 Ω, and 5000 Ω respectively. *R* values are recorded at the end of each iteration, along with the values of *V*_*prog*_, *I*_*lim*_, *V*_*meas*_*,* and *I*_*meas*_. The graphs are divided in grey/white zones, indicated in the top horizontal axis. These zones represent changes in the CBRAM cell resistance level, in the programming strategies and/or in the algorithm processes over time. The initial values of the algorithm variables are shown in Table 4 (see “Methods” section).

The algorithm operation for the *R*_*t*_ = 5 Ω experiment is plotted in Fig. 3a. The upper horizontal axis divides the programming process in different zones. Each zone refers to a specific behaviour of the programming operation. The whole process is detailed as follows. *Zone 1 ID* strategy is initially selected. *V*_*prog*_ progressively increases while *I*_*lim*_ stays still. The stationary resistance value implies that the CF is not being formed. During this interval, (1) applies. *Zone 2* the resistance suddenly drops to tens of kilo ohms. An abrupt increase in *I*_*meas*_ can be observed and *V*_*meas*_ cannot reach *V*_*prog*_, corresponding to the behaviour stated in (2). *Zone 3* a high current peak produces ion drifts within the dielectric which in turn produces filament connections and dissolutions. *Zone 4* a deep resistance drop occurs and *I*_*meas*_ reaches *I*_*lim*_ rapidly. *V*_*prog*_ cannot be applied. Instead, *V*_*meas*_ is the applied voltage (2). However, *R* > *R*_*t*_ and consequently the algorithm starts increasing *I*_*lim*_ to further decrease *R*. At this stage, the resistance is very low, just a few tens of ohms. Hence, the energy requirements for the cations to travel through the dielectric increase strongly. From second 13, the resistance value is below 10 Ω while the current and voltages keep increasing. *Zone 5* before reaching the target value, a high *I*_*meas*_ of 9.6 mA produces the rupture of the filament that increases *R* up to 539 kΩ. *Zone 6* as *V*_*prog*_ has not reached *V*_*max*_, it starts increasing from the previous value. *Zone 7* this time, due to higher electric fields together with the previously formed filament (zone 4), the reconstruction of the CF produces a bigger filament and therefore a lower cell resistance. *Zone 8 R*_*t*_ is achieved, and the stability check starts. At the end of the *sc*, *R* maintains a value under the target tolerance boundaries and the algorithm stops. Figure 3b–d show the algorithm operation for *R*_*t*_ = 50 Ω, 500 Ω and 5000 Ω, respectively. In the top horizontal axis, the zones are labelled using the acronyms of the strategies used during each period. The algorithm reaches the target in all cases (Fig. 3a–d). These results evince that the resistance tuning operation can be performed in both directions, i.e., using positive and negative voltages, or equivalently, increasing and decreasing the resistance. This is clear since every time the algorithm performs a stability check operation, it means that the measured resistance value falls into the desired boundaries. However, as the reader can observe in the graphs, the PRPA always finishes after an *ID* strategy followed by a stability check operation, which means that the conductive filament tends to be more stable after the application of a positive voltage pulse than after a negative one.

### DC performance

**Figure 4:**
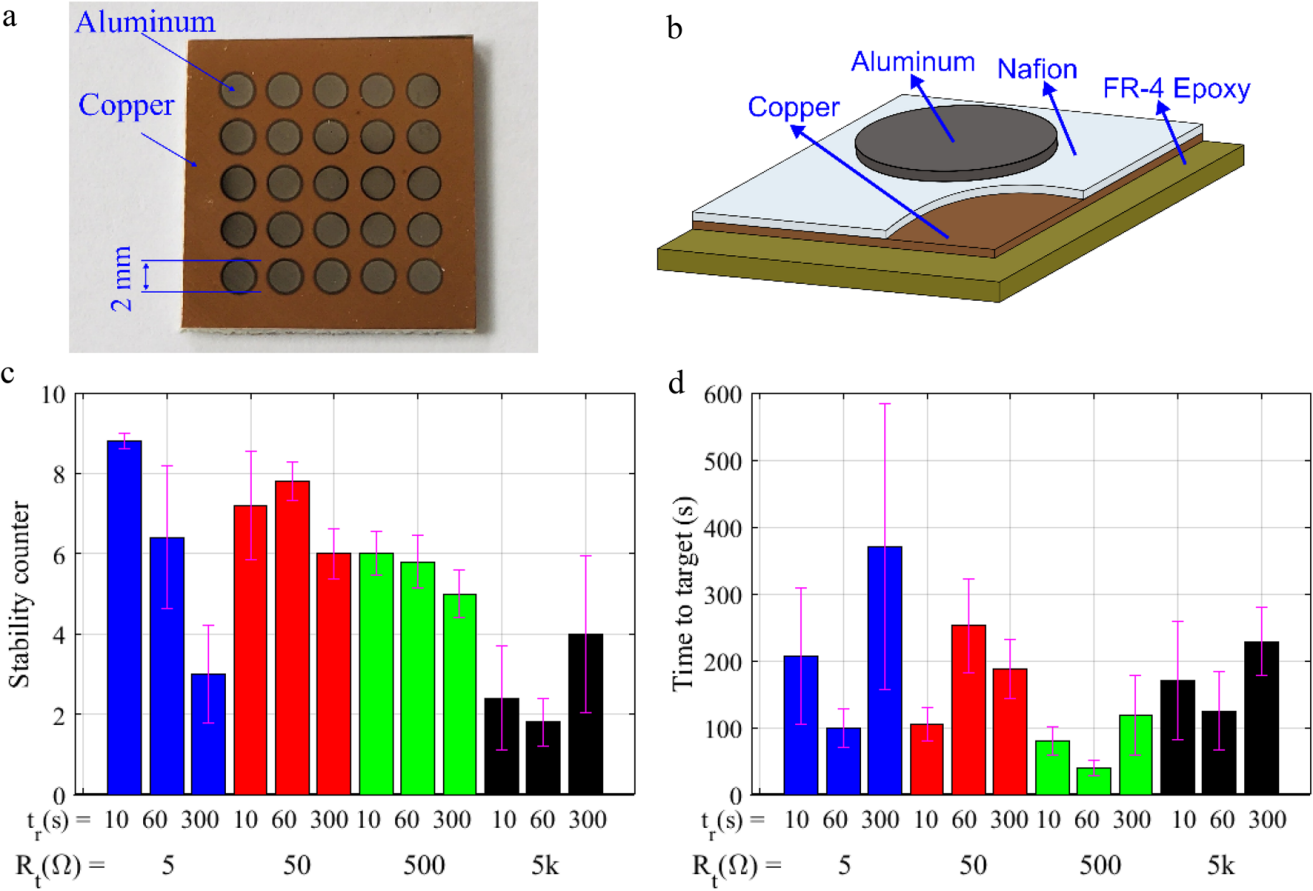
CBRAM switch cells on copper cladded FR-4 substrates used for DC characterization studies. (**a**) Photograph of fabricated CBRAM switch cell matrix. (**b**) Layer structure of a single cell of this group. DC performance tests over 60 different cells. (**c**) Stability results after programming virgin cells and (**d**) time to target for the tested target resistances, *R*_*t*_  = {5 Ω, 50 Ω, 500 Ω, 5000 Ω}.

In this experiment, we want to address the cell resistance stability after a successful programming operation using the PRPA. The experiment consists in running the algorithm over 60 different virgin CBRAM cells starting in a HRS. The cells are divided in four groups depending on their target resistance, *R*_*t*_ = {5 Ω, 50 Ω, 500 Ω, 5000 Ω}. Therefore, each group consists of 15 cells which are again divided by rest time in three groups, namely *t*_*r*_ = {10, 60 and 300 s}. For each cell, the algorithm is executed to program a single CBRAM cell. After the cell is programmed, it is left at rest for a period of *t*_*r*_ seconds and its resistance is measured again. Then, if the cell resistance is still within the tolerance limits of the target, the stability counter is increased in one unit, and the cell is again left at rest for another *t*_*r*_ s. Otherwise, the cell is reprogrammed using the PRPA. The rest-measure sequence is repeated nine times. Therefore, the cell resistance stability is measured from 0 to 9, 0 meaning that the cell was not able to retain the programmed value for any of the 9 measurements after the first programming operation, and 9 meaning that the cell resistance retained the programming value during the whole process. A photography of the realization of a batch of CBRAM cells is shown in Fig. 4a, and a scheme of the layer structure of a single cell is depicted in Fig. 4b.

The results of the experiment are illustrated in Fig. 4c,d. Figure 4c shows the mean and variance of the stability counter after the first programming operation executed on a virgin cell. Each bar represents the mean number of times a cell resistance is able to retain the programmed value within the target boundaries, for a specific *R*_*t*_ and *t*_*r*_. The results show a dependence of the stability with respect to *t*_*r*_. The most obvious case is for *R*_*t*_ = 5 Ω where the performance declines drastically with *t*_*r*_. However, looking at the first blue bar (*R*_*t*_ = 5 Ω and *t*_*r*_ = 10 s), the cell is able to maintain 4.5 Ω < *R* < 5.5 Ω for 900 s in almost all 5 realizations just by measuring the cell resistance every 10 s. In order to avoid fluctuations, the measurement pulses use *V*_*prog*_ = *V*_*meas*_ and *I*_*lim*_ = *I*_*meas*_ of the last pulse used during the programming operation. Looking back at Fig. 3, these tend to be low voltage pulses, consuming low power. Besides, we can observe a tendency towards decreasing the stability directly related with higher *R*_*t*_ values, which can be inferred also from Table 2. Table 2 shows the results when only *R*_*t*_, and not *t*_*r*_, is considered to calculate the mean and variance. From the results, it can be inferred that the best performance, in terms of stability, is achieved for *R*_*t*_ between 5 and 500 Ω.

**Table 2:** Statistic results per *R*_*t*_.

	Mean/variance			
*R*_*t*_ (Ω)	5	50	500	5000
Number of stable measurements	6.06/0.92	7/0.52	5.86/0.42	2.46/0.76
Mean time to target (s)	225.36/53.85	181.45/13.22	76.75/15.45	160.80/11.34

The other figure of merit assessed in this experiment was the time required for the PRPA to perform a successful programming operation, also referred as the time to target. Figure 4d shows the mean and variance of the time to target obtained from all PRPA executions during this experiment. As expected, reaching the desired accuracy for low resistance values (ε < 1 Ω if *R*_*t*_ < 10 Ω) requires a high number of iterations leading to the longest algorithm execution times. The best performance in terms of time to reach the target was between 50 and 5 kΩ.

### RF switch

**Figure 5:**
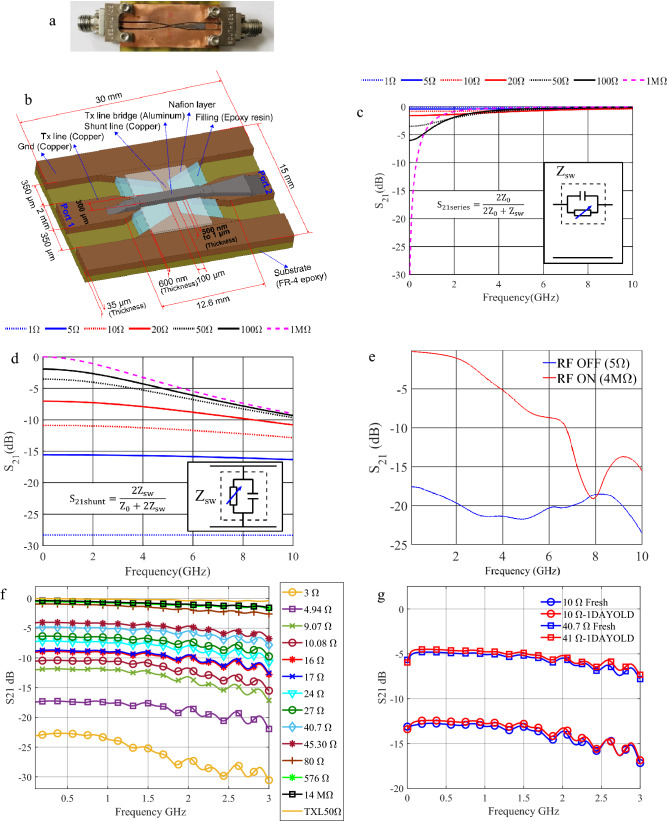
CBRAM based CPW shunt mode RF switch programmed using the proposed PRPA algorithm. (**a**) Photograph of fabricated device. (**b**) Topology and layer structure. (**c**) S_21_ parameter model values of the CBRAM cell connected in series to a transmission line of characteristic impedance of Z_0_ = 50 Ω. (**d**) S_21_ parameter model values of the CBRAM cell connected in parallel (shunt) to a transmission line of characteristic impedance of Z_0_ = 50 Ω. The CBRAM cell has been modelled as a shunt RC circuit of impedance Z_SW_ in both cases (**c,d**) with C = 1681 fF. (**e**) Simulation of the S_21_ parameter of the presented RF switch up to 20 GHz for ON and OFF states. (**f**) Measured RF transmission characteristics. (**g**) Measured time stability of the RF transmission.

In this section, we present the application of the PRPA on a CBRAM based RF switch to test its effectivity for electronically controlled RF attenuator applications. The proposed device consists of a CPW based shunt mode RF switch constructed by integrating a CBRAM cell on the CPW transmission line geometry^6^. A reproduction of this CPW shunt mode RF switch used for the experiments described in this article is given in Fig. 5a together with a schematic of the switch design (Fig. 5b). The shunt configuration presents two main advantages with respect to the series one. Namely, providing control over a wide range of values of the S_21_ parameter, using the moderate low LRS (of the order of 1 to 100 Ω), and mitigating the bandwidth restrictions imposed by the high OFF state capacitance (≅ 1 pF in the presented case). To give more insight into these assertions, we introduce the electrical models^9^ of both series and shunt configurations in Fig. 5c,d respectively. As described in Fig. 1b, the CBRAM cell has been modelled as a shunt RC circuit of impedance Z_SW_ in both cases. Looking at the series configurations (Fig. 5c), we can observe that the attenuator dynamic range decreases drastically as the frequency increases. In other words, the usable bandwidth is constrained by the desired attenuation range. For instance, the device bandwidth is less than 2 GHz for an attenuation range of 0–5 dB. In the same way, the S_21_ values change abruptly for short frequency intervals, which makes the high resistance values not reliable to obtain stable attenuations. On the other hand, the shunt configuration (Fig. 5d) shows a much better behaviour in terms of bandwidth and it presents a flat band up to 3 GHz, for the whole dynamic range of the attenuator.

The simulation results of the device response (S_21_) for ON and OFF switching states are displayed in Fig. 5e. The results show that the switch tends to deviate from a desired insertion loss at frequencies beyond 3 GHz. This is due to the high OFF-state capacitance of the CBRAM cell. This limitation of the frequency band comes mainly from the methods of realization that we privilege: realization outside of clean room with common materials (no silicon wafer for example), even flexible.

In this experiment, several arbitrary resistance values selected in a random manner are presented to the PRPA and the resulting modulated waveform is fed to the CBRAM cell of the RF switch. Eventually the outcome of this operation is that either the CBRAM switch is programmed to the desired resistance state within the tolerance boundaries, or the algorithm is terminated on meeting the time limit. Figure 5c shows the transmission characteristics of the CBRAM based RF switch for successful programming operations, latched to the desired resistance state. Among the transmission parameters of the CPW shunt mode switch, one of the traces indicates the transmission characteristics of a regular transmission line of length 30 mm and characteristic impedance 50 Ω, using the same calibration as applied to the vector network analyzer (VNA) for the measurement of the RF switch. This trace (noted TXL50 Ω in Fig. 5c) is given here as a reference to filter out the calibration error and effects of port cables and feed connectors used with the RF switch which is being studied. The range between 5 and 100 Ω are the values that exhibit notable controllable RF attenuation characteristics among the ON and OFF states of the RF switch under consideration.

The impedance stability over time of the CPW shunt mode RF switch was tested for two random impedance states over an interval of about 24 h or 86,400 s. For this, the switch was programmed to a custom chosen resistance and was left in ambient environment after recording the RF transmission characteristics as well as the impedance state of the integrated CBRAM cell. This switch was again re-measured, for the RF transmission and impedance characteristics, after the chosen time limit and was found to be stable with notable agreement among the starting and final observations. These observations are depicted in Fig. 5d. Slight variations in the RF transmission characteristics of the order of around 0.5 dB or less are justified by calibration errors, effects of connecting port cables and electro-migration over time.

## Discussion

The results presented in the previous section support the proposed algorithm as an effective tool for the resistance programing of RF CBRAM cells to the desired target resistance with an error smaller than 10%. This means that for a low value of 5 Ω, the tolerance is only ± 0.5 Ω, instead of a 1% tolerance value of ± 50 Ω for a CBRAM memory device with an LRS of 5 kΩ. The PRPA has been tested over a wide range of target resistances, and the cells were programmed successfully in a limited amount of time. In addition, results from the DC performance experiment show that readouts can be destructive and a detailed discussion about this issue can be found in the Supplementary Discussion.

Through this study, we show that the link between the geometry of the filaments and the voltages/currents of activation can be used in practice for the realization of new components. The programming voltage/current applied on the CBRAM allows modifying its constitution and thus its resistance, and at the same time, we observe a sufficient repeatability of this dependence which allows, using an iterative algorithm, to impose a pre-determined resistance to the CBRAM cell. However, it is noted that even though retention time is limited, it is possible to introduce occasional refresh. Again, in this case, it will be possible to cut down the total power consumption by several folds than as required with a classic volatile RF switch.

The presented results support the feasibility of the resistance control of CBRAM cells, which can have a strong impact in the development of electronically programmable RF devices. The above experiment shows that the proposed PRPA algorithm allows controlling the RF transmission through the resistance control of a CBRAM cell, facilitating the electronic reconfigurability of RF and microwave devices. A particularly expected result is to realize a non-volatile variable RF attenuator based on the CBRAM technology.

This switch, which is essentially a non-volatile device, could be easily converted to an electronically programmable RF attenuator, constructed in a desired configuration (e.g., ‘T’, ‘π’, or hybrid topologies). Even though the commercial attenuators available on the market provide a very high bandwidth (at least 10 times larger than the presented bandwidth) and very high attenuation levels (up to 120 or 150 dB), there are not any non-volatile solid-state electronically programmable attenuators commercially available at present, and the proof of concept presented in this article is the first one of its kind, to the best of our knowledge and belief. Besides, the results for the predicted behaviour of the switch beyond 3 GHz, presented in Fig. 5e, have an important implication. From them, we can conclude that the parallel capacitance is a critical parameter for RF switches when comparing them to simple memory cells. This parasitic component is usually not discussed in the state of the art, specially, in those contributions focused on memories.

For the sake of comparison with the current state of the art, Table 3 includes the best performance CBRAM RF switches found in a thorough review of the literature^5^. The performance in the operating band of the presented switch is comparable to the literature, even achieving lower R_ON_ (2 Ω) than other works.

**Table 3:** A comparison between the performance obtained with our RF switch and the state of the art in the field of CBRAM RF switch.

Ref.	Technology	IL (dB)	IS (dB)	Bandwidth (GHz)	R_ON_ (Ω)	C_OFF_ (fF)	FOM (THz)	Area (µm^2^)	Actuation voltage (V)	Power handling (W)	Endurance (#cycles)
This	CBRAM	< 1.1	> 25	DC-3	2	1681	0.051	Π × 10^6^	16	N/A	> 10^3^
^8^	CBRAM	< 0.5	> 35	DC-6	6	9	2.95	400	1	0.5	N/A
^9^	CBRAM	< 0.3	> 30	DC-40	2.6	1.45	42.2	0.6	3	0.1	< 10^3^
^10^	2DM CBRAM	< 0.25	> 29	DC-67	2.7	0.84	70	0.03	1	N/A	> 10^3^
^11^	2DM CBRAM	< 0.2	> 15	DC-110	1.6	2.3	43	0.25	1.5	0.1	N/A

## Methods

### Fabrication techniques

The CBRAM switching cell matrix used for DC characterization of the developed precision resistance programming algorithm is shown in Fig. 4a. The fabrication process of these cells was reported in Ref.^13^, and is detailed next. The cells are fabricated on a commercial copper cladded FR-4 substrate, with adequate copper layer thickness (say ~ 35 μm) to ensure mechanical and electrical stability. Towards fabrication, the copper surface is first cleaned with acetone to remove any impurities and/or oxide layers. This layer forms the active electrode of the CBRAM cells, followed by spin coating of Nafion resin solution^27^ at a pre-set rate of 500 rpm, for 30 s, to achieve a final layer thickness of 600 nm. The substrate is then either dried in ambient environment for a few hours, or baked on a hot plate at 45 °C, for 60 s for fast drying. Then, circular pad shaped aluminium inert electrode layers are deposited with thermal vapor deposition technique with the aid of a dedicated nickel mask with adequate aperture size. The deposited aluminium layer has thickness of the order of 500 nm–1 μm, and a diameter of 2 mm as shown in Fig. 4a.

The realization and topology of the CBRAM based CPW shunt mode RF switch is depicted in Fig. 5a. This is a shunt mode switch constructed by integrating a CBRAM cell by adapting the geometries of a CPW transmission line. Here the two ground planes of the CPW line are connected among each other, at the center of the line, with a 100 µm wide shunt line as shown in Fig. 5b, and a 600 nm thick layer of Nafion (ion-conductor) sandwiched among this (copper-active electrode) shunt line and a 300 µm wide aluminium metallization (inert electrode) that connects the tapered transmission line segments, connecting the input–output ports, which completes the integration of the CBRAM switch to this line. The realization steps towards this structure are as follows. First, the CPW transmission structures in copper are engraved on a copper cladded FR-4 substrate through standard PCB-photolithography, then an epoxy resin compound is applied to the CPW gap, at the region where the CBRAM switch is formed and then mechanically polished to a mirror finish (with a surface roughness typically less than around 100 nm), to obtain a smooth surface for the deposit of the Nafion layer as shown in Fig. 5b. Following this step, the Nafion layer is formed by spin coating of the Nafion resin solution and air-drying/baking, as explained in the case of the switch cell matrix used for DC characterization. Then, the final segment (aluminium inert electrode), connecting the two transmission line structures (connecting port 1 and 2) is deposited by thermal vapor deposition through a nickel mask with appropriately engraved aperture. The detailed fabrication process of this switch, along with the explanation of the relevance of each layer present is given in Ref.^6^.

### Measurement setup

The synthesized switching wave form generation is performed by the source measure units (SMU), Keithley 2450 and 2400^28^ respectively for the CBRAM switch matrix and CPW-CBRAM RF switch, controlled by the PRPA.

In case of the CBRAM switch matrix, the programming waveform is applied to the switches through a pair of custom-made probes, housed on an in-house made probing platform. The resistance of the cables and probes are de-embedded from the programming path, by short-circuiting the probes on a polished copper surface, and subtracting this resistance value from the programming path, while the measurements are done on CBRAM cells.

Similarly, the programming pulses are applied to the RF switch through the SMA coaxial connectors, using a coaxial cable of appropriate gender connected to it. Any RF connections to the switch are disconnected during this process. The above said resistance de-embedding is applied in this case as well, by shorting the DC pulse feed cable on a short circuited SMA connector block. Agilent ENA E 5061B 3 GHz VNA^29^ is used to record the RF transmission characteristics of the RF switch under analysis. The VNA is calibrated till the RF-port cable ends, with standard short-open-load-through (SOLT) 2-port calibration. An S-Parameter measurement is done every time a resistance state is finalized, and the CBRAM resistance state is re-measured immediately after the RF measurement to confirm that it is unchanged, to make sure the recorded data is stable.

### PRPA pseudocode

The pseudocode containing the main procedures of the PRPA is exhibited in Alg. 1 and Alg. 2. The algorithm takes the target resistance *R*_*t*_ as the input parameter. The cell is successfully programmed when *R* falls between the boundaries of the interval [*R*_*min*1_, *R*_*max*1_], where *R*_*min*1_ = (1 − *tol*_1_) *R*_*t*_ and *R*_*max*1_ = (1 + *tol*_1_) *R*_*t*_, and *tol*_1_ is the tolerance with respect to the target value. While a different interval *R* ∈ [*R*_*min*2_, *R*_*max*2_], *R*_*min*2_ = (1 − *tol*_2_) *R*_*t*_ and *R*_*max*2_ = (1 + *tol*_2_) *R*_*t*_ can be used to stop the *ID* procedure at a specific threshold and modify the parameter updating rules in order to slow down the resistance decreasing process. This has been only required for *R*_*t*_ values higher than 1 kΩ. tol_2_ is again the tolerance with respect to the target value. In the case of 5 kΩ, *tol*_2_ was set to 90%.

The input parameters are updated during the execution of the *ID* and *II* procedures according to the following. The update function is applied to both input parameters, *V*_*prog*_ and *I*_*lim*_, and it consists of a percentage increase of the past values. Specifically, *V*_*prog*_ is updated when *V*_*meas*_ is close to *V*_*prog*_. *V*_*th*_ is the factor that accounts for this proximity (Alg. 2, lines 5 and 12). While *I*_*lim*_ is updated when its value is equal to *I*_*meas*_. *V*_*inc*_ and *I*_*inc*_ correspond to the voltage and current percentage variation respectively. In this way, the magnitude variations, of both input parameters, are small for low values, and they progressively increase for higher ones. The authors have no doubt that the update operation is a critical one. Thus, it could be modified to further improve the performance of the algorithm by selecting more adaptive operations, though this may require taking into account other variables, like the electrolyte and/or electrodes properties.

Here, *state* indicates whether the previous cell resistance value is higher or lower than the target, i.e., *R* > *R*_*t*_ (*state* = 1) or *R* < *R*_*t*_ (*state* = 2), so that if the previous and the current state differ, the polarity of the programming pulse has to be inverted and the variables are updated according to the new state initial values.

*V*_+_, *V*_*−*_ and *I*_±_ are designed to safely operate the CBRAM cells with direct (+) and inverse (−) polarity. Here, *strategy* indicates the selected procedure (Alg. 2), *mode* controls the parameters updating process (*V*_*prog*_, *I*_*lim*_) in the *ID* procedure in order to induce a smoother approach to the target resistance, for high *R*_*t*_ values greater than 1 kΩ. This way the algorithm will try to slow down the filament build-up before *R* gets close to *R*_*t*_. τ_*tri*_ is the duration of the triangular voltage pulse used in the *TP* procedure, and τ_*rect*_ is the duration of the rectangular pulses used in *ID*, *RP*, *II* and *sc* procedures. The initial values of all the other involved variables are presented in Table 4.

**Table 4:** PRPA variables.

*V*_+_	12 V	*V*_*th*_	10%
*V*_*−*_	− 20 V	*V*_*inc*_	10%
*V*_*prog*_	V_0+_	*I*_*inc*_	10%
*V*_*tri*_	16 V	*tol*_1_	10%
V_0±_	± 1 V	*tol*_2_	10%
*I*_±_	± 0.2 A	*Mode*	1
*I*_*lim*_	I_0±_	*State*	1
I_0+_	500 μA	*Strategy*	1
I_0−_	− 100 μA	*τ*_*tri*_	12 s
		*τ*_*rect*_	0.18 s

## Supplementary Information

Supplementary Information.

## Data Availability

The algorithm code can be requested to S.L.S.
