# Supplementary material for: CBRAM technology: transition from a memory cell to a programmable and non-volatile impedance for new radiofrequency applications
Source: Sci Rep. 2022 Mar 8;12:4105. doi: 10.1038/s41598-022-08127-x (PMC8904552; doi:10.1038/s41598-022-08127-x)
Supplement: Supplementary file 1 — Supplementary Information. [file 41598_2022_8127_MOESM1_ESM.docx]

CBRAM Technology: Transition from a Memory Cell to a Programmable and Non-Volatile Impedance for New Radiofrequency Applications

**Supplementary Information**

# Supplementary note 1.

**CBRAM cell performance.** The characteristic I-V curve of the implemented devices is illustrated in Supplementary Fig. 1**.**

| 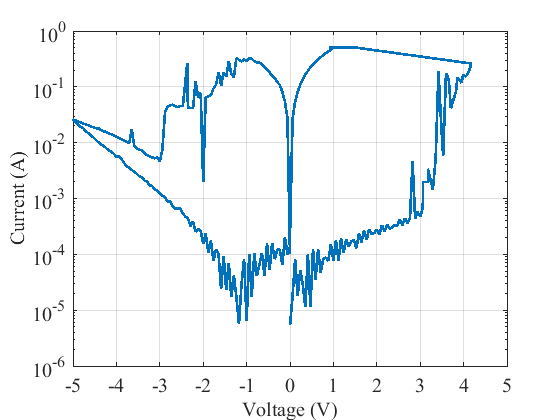 |
| --- |
| Supplementary Fig. 1 **\| Switching I-V curve showing the switching cycles to LRS and HRS. This measurement was performed over a virgin cell fabricated under the same procedures than the cells of Fig. 4a of the article.** |

# Supplementary note 2.

**Set, unipolar and bipolar reset examples.** A set operation in two stairs can be seen at the beginning of the programming cycle in Supplementary Fig. 2a. The set operation is detailed next. *R* starts in the order of MΩ while *V_meas_* = *V_prog_* increases. In t = 6 s, *R* drops to 100 kΩ while *I_meas_* = *I_lim_*. At t = 9 s, *R* falls below 100 Ω. The programming algorithm continues until the control loop exit condition is satisfied (Fig. 2b).

An example of unipolar reset can also be found in Supplementary Fig. 2a. After the initial set, the cell resistance is 10 Ω < *R* < 100 Ω, while *V_meas_* starts increasing and *V_prog_* = 5 V. *I_meas_* and *I_lim_* are also increasing until a unipolar reset is triggered at t ≈ 25 s.

Precisely controlled bipolar reset cycles can be observed in Supplementary Fig. 2b at several instants during the programming execution at t = 61, 94 and 130 s. The opposite polarity is generated through negative values of *V_meas_*.

# Supplementary Discussion.

**Destructive readouts.** Supplementary Fig. 2a-d show the experiments carried out over single cells during the DC performance subsection of the main document. Each figure corresponds to a full experiment on a single cell. As explained in the main manuscript, it consists of an initial programming execution followed by 9 check rounds. The time between programming and the next check round and also the time between check rounds is *tr*. *tr* intervals have been coloured in grey in Supplementary Fig. 2a-d. In the following examples *tr* = 10 s. If after each check round the cell resistance falls out of the target resistance boundaries the programming operation is triggered. The target resistance boundaries are illustrated as horizontal black lines. Supplementary Fig. 2a-d represent the full experiment corresponding to the programming cycles of Fig. 3a-d of the main document respectively.

The readout pulses used for round checks during these experiments were 1 V and 500 µA. It is worth noticing that these pulses were sometimes destructive and further analysis of this issue is required. The future work involves using the last values applied to the cell at the end of the programming cycles as readout values, since these values tend to be low voltage and current values, as the reader can appreciate from Fig. 3a-d of the main document.

The current results provide more information about this issue as we are going to see next. The examples of Supplementary Fig. 2a and Supplementary Fig. 2b show good performance keeping the cell resistance inside the target region 9 out of 9 round checks. However, Supplementary Fig. 2c only keeps the value 6 out of 9 round checks, while Supplementary Fig. 2d cannot retain the programmed value after any of the round checks.

In the case of Supplementary Fig. 2c, the slight deviations of the cell resistance from the target boundaries produce new programming cycles. This deviation, resultant from the inability of the conductive filament to maintain its structure, can be caused by a high temperature of the electrolyte and other effects different than destructive readouts. However, Supplementary Fig. 2d shows how the readout voltage of 1 V can be applied at all check rounds. The reason is that equation (1) is met due to the high cell resistance, which makes the readout to be destructive and the round check to fail.

The main conclusion extracted from these results is that the retention could be much higher than the observed one in this study if the readouts were less destructive. And thus, this issue will be the focus of future works.

| a  R  R  t  I  m  e  a  s  I  l  i  m  V  m  e  a  s  V  p  r  o  g |
| --- |
| 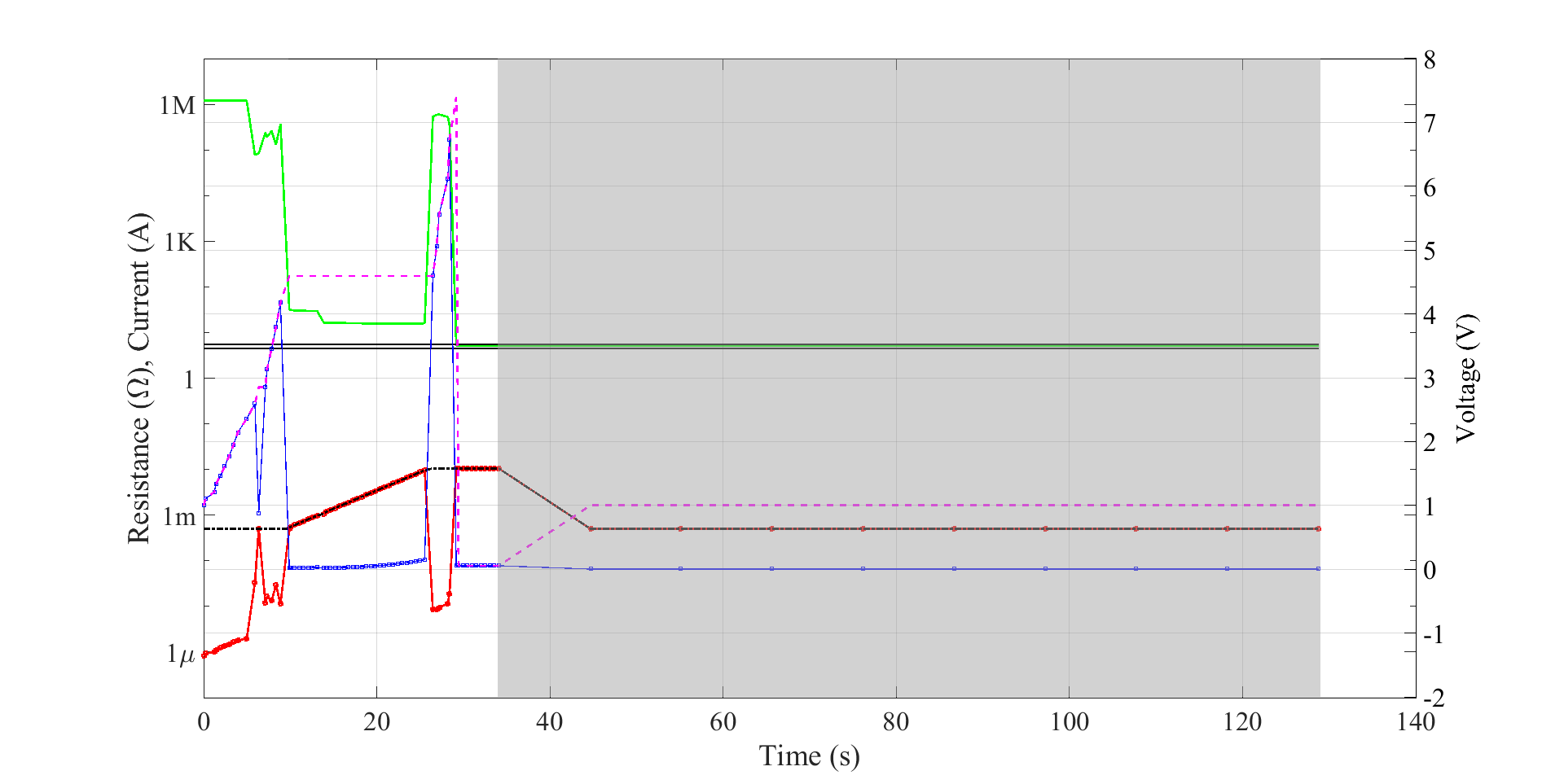 |
| b  R  R  t  I  m  e  a  s  I  l  i  m  V  m  e  a  s  V  p  r  o  g |
| 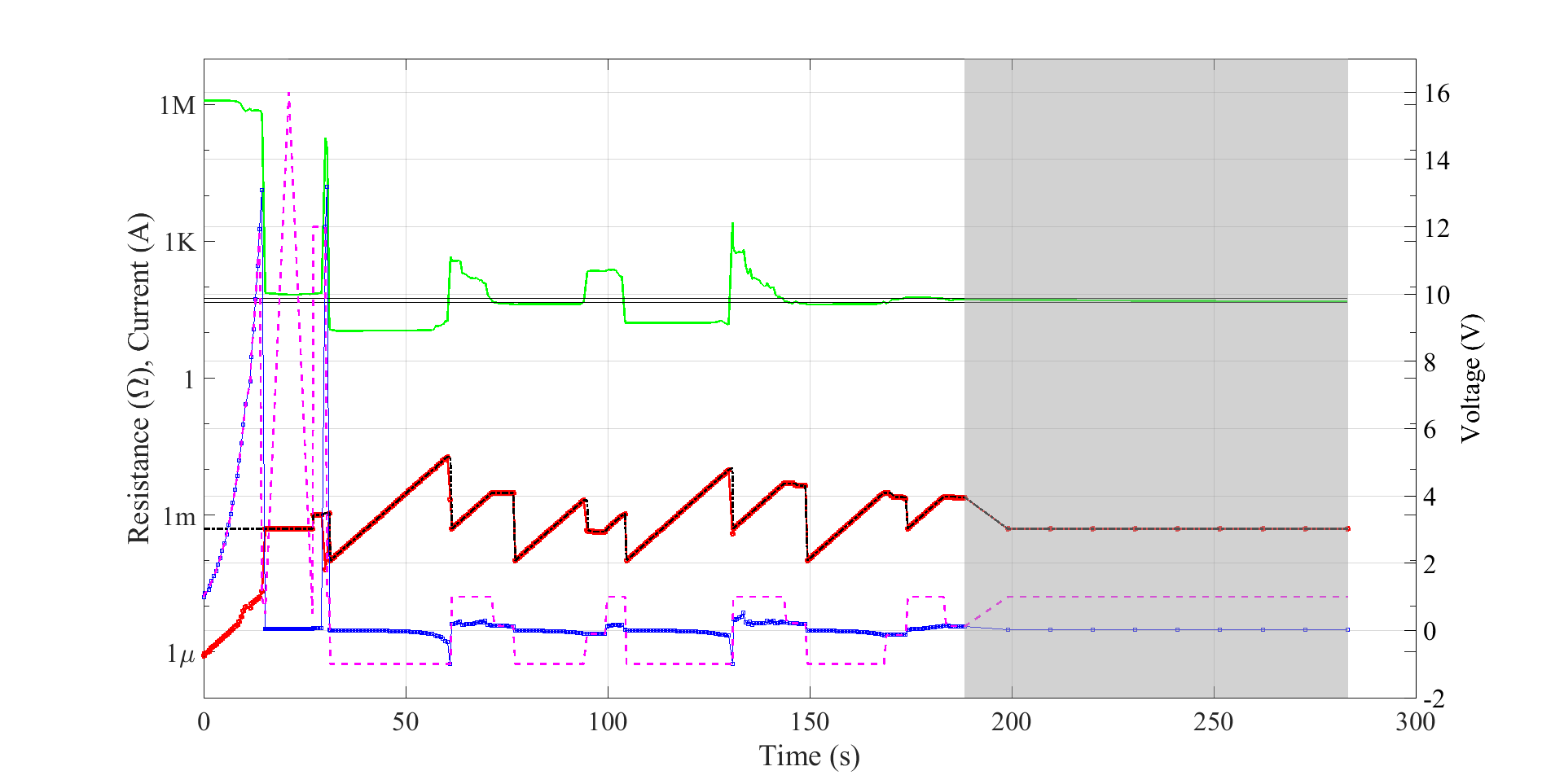 |
| R  R  t  I  m  e  a  s  I  l  i  m  V  m  e  a  s  V  p  r  o  g |
| c 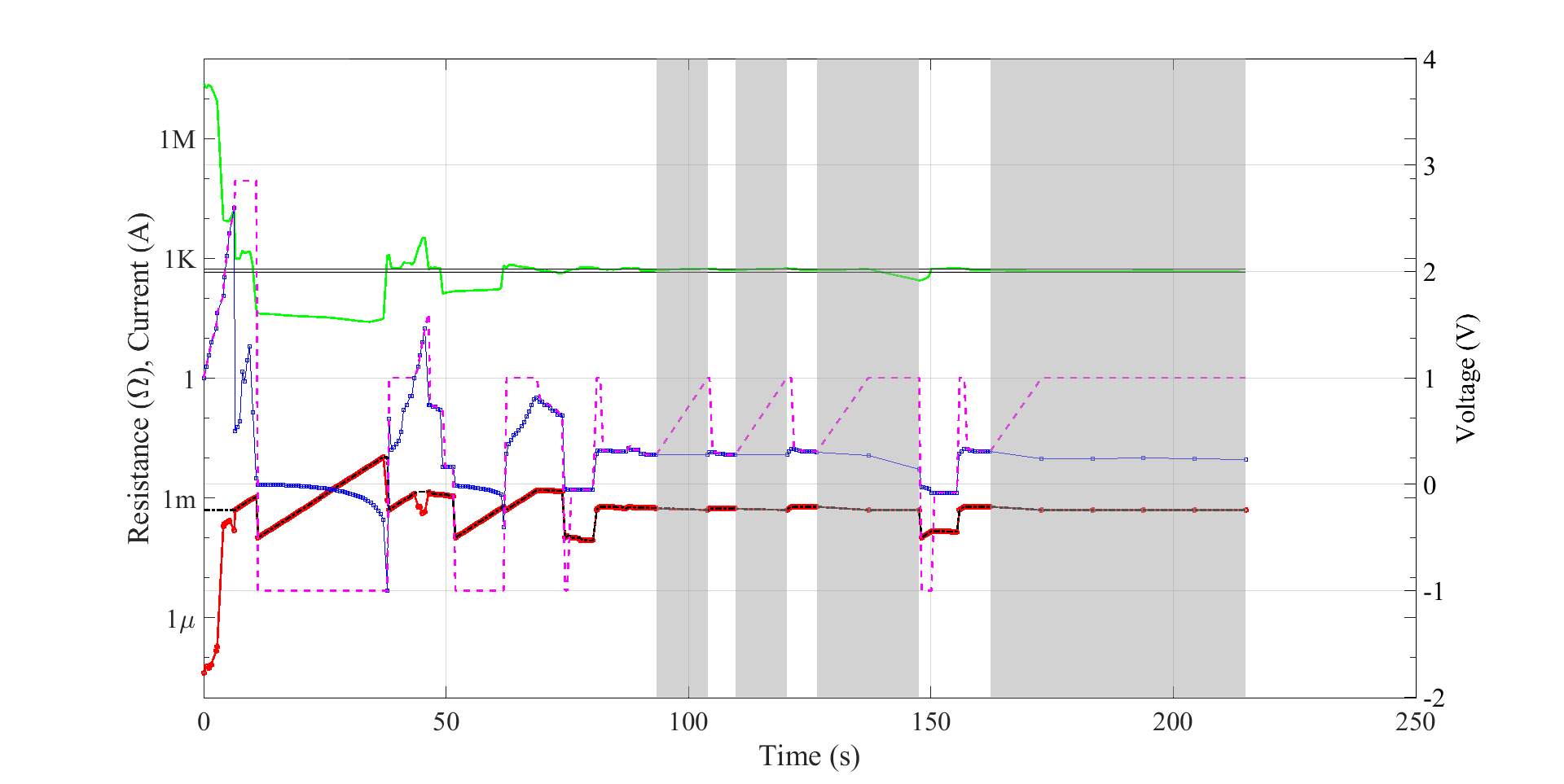 Successful round check  Unsuccessful round check |
| R  R  t  I  m  e  a  s  I  l  i  m  V  m  e  a  s  V  p  r  o  g |
| d 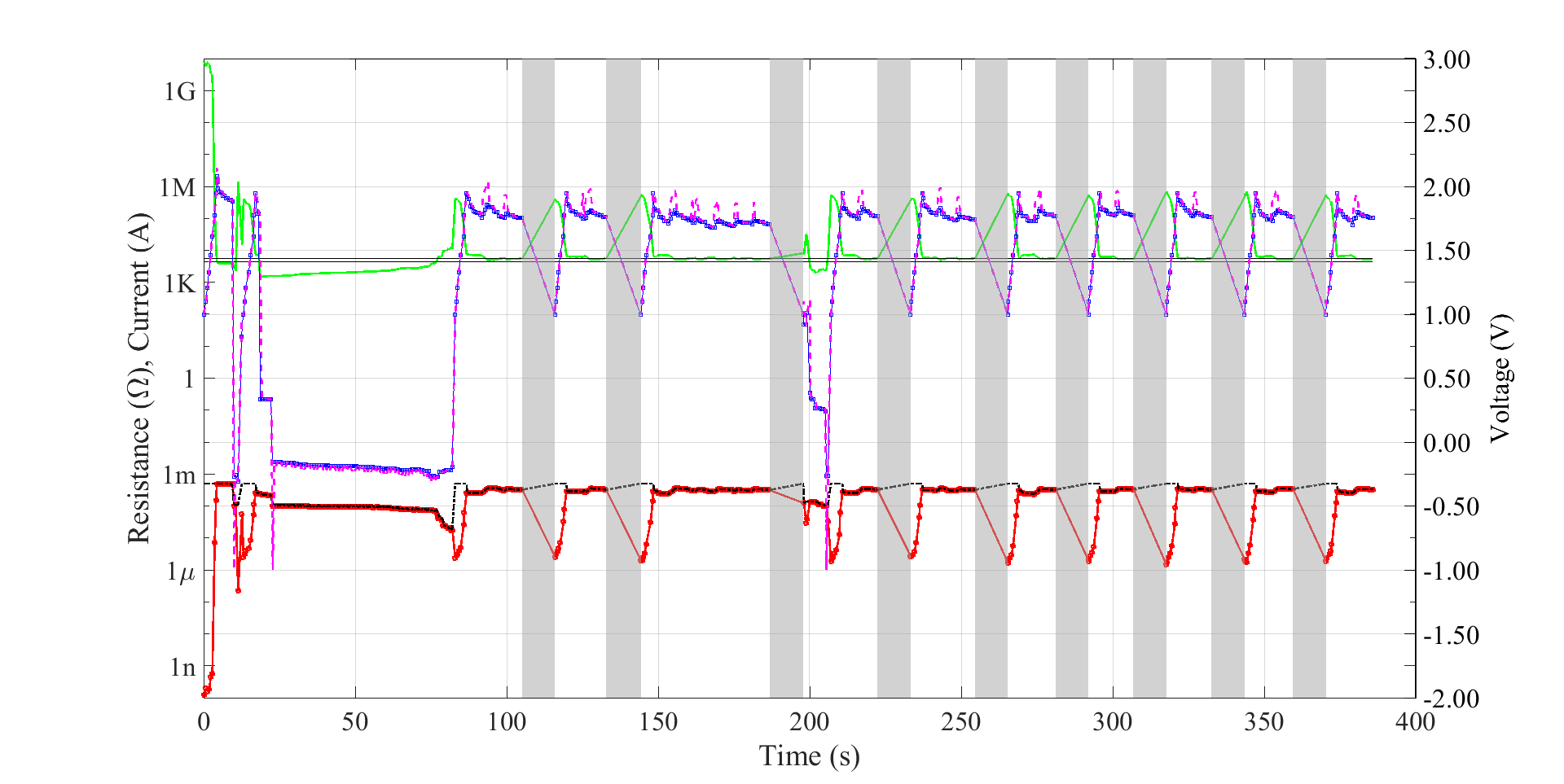 |
| Supplementary Fig. 2 **\| Full DC performance experiment realised on an individual cell with *tr* = 10 s and (a) *Rt* = 5 Ω. (b) *Rt* = 50 Ω. (c) *Rt* = 500 Ω. (d) *Rt* = 5000 Ω.** |
